# Left sided inferior vena cava duplication and venous thromboembolism: case report and review of literature

**DOI:** 10.1186/1756-8722-1-24

**Published:** 2008-12-02

**Authors:** Cannon Milani, Maria Constantinou, David Berz, James N Butera, Gerald A Colvin

**Affiliations:** 1 Warren Alpert School of Medicine at Brown University and Department of Medicine, Rhode Island Hospital, Providence, RI, USA

## Abstract

The etiology of venous thromboembolism in young patients is frequently associated with hereditary coagulation abnormalities, immunologic diseases, and neoplasia. The advent of radiological advances, namely Computed Tomography (CT) scans and venography has identified vena cava malformations as a new etiologic factor worthy of consideration. In this case report, we describe the unusual occurrence of venous thromboembolism in association with a duplicated inferior vena cava. Duplications of the inferior vena cava (IVC) are seen with an incidence of 0.2% to 3.0% in the general population. Embryogenesis of the IVC is a complex process involving the intricate formation and regression of numerous anastomoses, potentially leading to various anomalies. We present a 23-year-old Caucasian woman with IVC duplication who developed a deep venous thrombosis and multiple pulmonary emboli. Anomaly of the IVC is a rare example of a congenital condition that predisposes to thromboembolism, presumably by favoring venous stasis. This diagnosis should be considered in patients under the age of 30 with spontaneous occurrence of blood clots.

## Background

Over a century ago the German physician, Rudolf Virchow, was credited for elucidating the mechanism of pulmonary thromboembolism. The factors contributing to venous thrombosis came to be known as Virchow's triad. The three components were 'abnormalities of blood vessel wall', 'abnormalities of blood constituents', and 'abnormalities of blood flow'. [1]

The incidence of deep venous thrombosis (DVT) in western populations is estimated at 1 in 1,000 individuals per annum. [2] This figure varies with age, and in adults aged between 20 and 40 years, the incidence is 10 times lower. [3]

The objective of this case report is to describe IVC malformation in a young patient as a risk factor in the development of venous thromboembolism and the associated clinical manifestations. This case provides the opportunity to review the dysregulated embryogenesis of this phenomenon, and the intricate management questions that arise.

**Figure 1:**
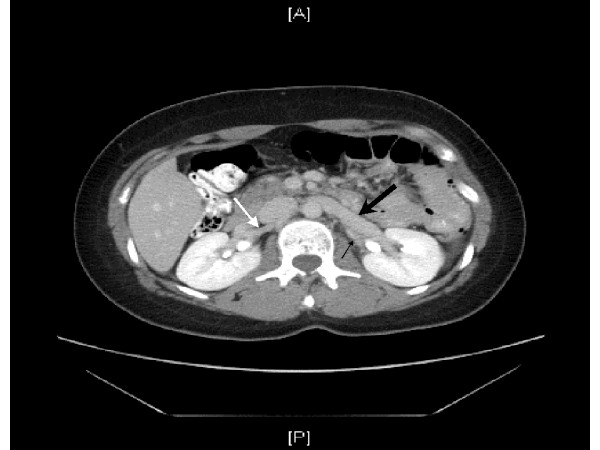
The left sided IVC (thin black arrow) is shown joining the left renal vein (thick black arrow). At the same level the right renal artery (white arrow) is visible

In duplication of the IVC, there is a normal inferior vena cava along the right side of the spine. In addition, a left-sided IVC ascends to the level of the renal veins to join the right-sided IVC through a vascular structure that may pass either anterior or posterior to the aorta at the level of the renal veins. The characteristic CT appearance (Figure 1) is a single right-sided inferior vena cava at levels above the renal veins, a vascular structure crossing either anterior or posterior to the aorta at the level of the renal veins, and a vascular structure to the left (i.e. the duplicated IVC) and right of the aorta below the level of the renal veins. [4]

## Case Presentation

Our 23-year-old female patient with no significant past medical history presented to the hospital complaining of acute shortness of breath, abdominal pain, and severe left lower extremity pain. The patient was in her usual state of health until the evening of her symptoms, when she suddenly turned in her bed and began to experience numbness, tingling, and purplish discoloration of her left lower extremity. On arrival to the emergency department, the patient was in respiratory distress and had bilateral swelling of her lower extremities, the left side larger than the right. A bilateral lower extremity duplex ultrasound revealed a deep vein thrombosis (DVT) in the left lower extremity, the popliteal, and the femoral veins. In addition a CT of the chest utilizing our pulmonary embolism protocol delineated two pulmonary emboli in the first order pulmonary arteries and multiple segmental emboli located in the right upper and right lower lobes. She was subsequently started on a heparin drip.

The patient denied any tobacco use, recent immobilization, or travel. She reported the use of an estrogen vaginal delivery device (ethinyl estradiol vaginal ring) for the previous 6 months. Her hypercoagulable workup was revealing for heterozygosity at C677T for factor V Leiden. It should be noted that heterozygous carriers of FVL have been shown to have an overall 3- to 7-fold increased risk of venous thrombosis, while homozygotes to C677T have a 50- to 100-fold increased risk. [5]

Due to the acute presentation of her venous thromboembolism, a left pelvic and lower extremity venous thrombectomy was performed. This was followed by stenting of a popliteal venous occlusion, and subsequent thrombolysis with tissue plasminogen activator. Surprisingly, a left lower extremity venogram revealed bilateral inferior vena cavas.

**Figure 2:**
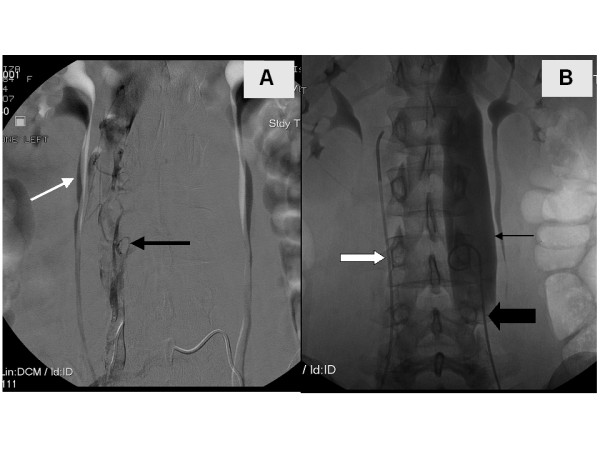
**A: **Patient in prone position delineating the stippled appearance of the left duplicated IVC (black arrow) in communication with the left renal vein (white arrow). **2B: **Patient in supine position with balloon angioplasty of the duplicated left IVC (thin black arrow) and iliac stent (thick black arrow), with improved appearance and flow through the stents. Simultaneously the right IVC undergoing catheterization (white arrow).

**Figure 3:**
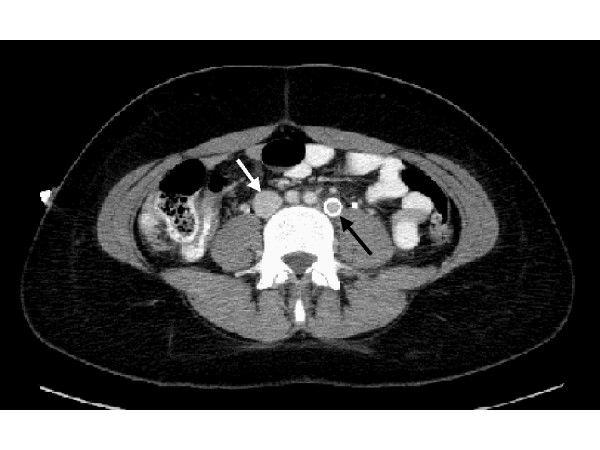
Duplicated left IVC with wall-stent draining into the left renal vein (black arrow). There is a non-occlusive thrombus within it. The orthotopic IVC (white arrow) appears widely patent.

In our patient, the left-sided duplicated IVC and iliac veins were found to be thrombosed. This was marked by the stippled appearance of the duplicated left IVC vessel seen in Figure 2a. The decision was made to stent the duplicated IVC given the anatomic irregularity and increased risk of repeat thrombosis. Two wall stents (Figure 2b) were deployed at the proximal end at the level of the left sided (duplicated) IVC. Next a balloon was used to angioplasty the entire length of the stented segment of the duplicated IVC (Figure 2b). Figure 3 depicts the duplicated left IVC with wall-stent draining into the left renal vein with a non-occlusive thrombus within it. The orthotopic IVC remained patent. The patient was kept on heparin for 24-hours and then started on Coumadin.

## Discussion and Conclusion

There have been few case reports with IVC anomalies and development of deep vein thrombosis. In our comprehensive English language literature review, the earliest reported case of duplication of the inferior vena cava was reported in 1912 in the journal, The Anatomical Record. [6] Over the last 100 years, case series involving IVC duplication in association with venous thromboembolism number less than 10.

This observation was made following an extensive PubMed literature search encompassing the following keywords: inferior vena cava, congenital, deep venous thrombosis, and pulmonary embolism. From an anatomical standpoint in adults, IVC has 3 segments of different embryologic origin: pre-renal, renal, and post-renal. This type of fusion and partial re-absorption of three pairs of vessels is dependent upon the posterior cardinal veins in the embryo. This complicated evolutionary process can give rise to anatomic malformations that impede vein drainage and favor the development of thrombosis. [7,8]

Duplication of the IVC, the prominent manifestation in our case report, occurs because the left supracardinal vein fails to regress early in gestation resulting in large veins on both sides of the Aorta that usually joins anterior to the level of the renal arteries to become the suprarenal IVC. [9] When incidentally found, treatment options include observation, placing filters in either systems, or coil-embolization of the duplicated segment plus placing a filter in the right IVC.

**Figure 4:**
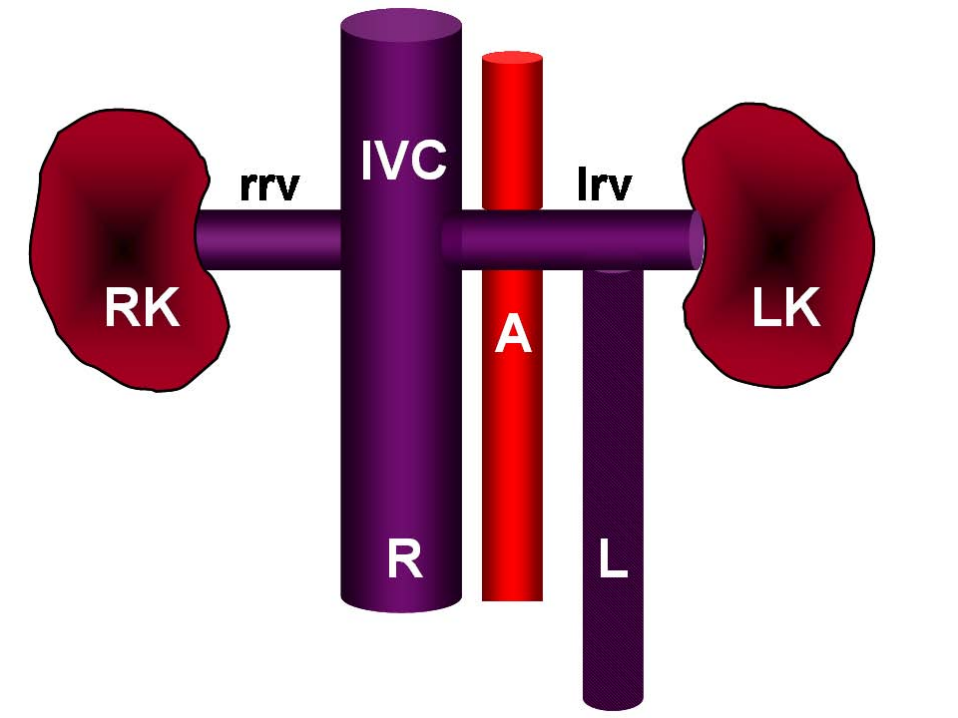
Diagram of the duplicated IVC of our patient based on ultrasound showing the right and left kidneys (RK and LK), the aorta (A), the right renal vein (rrv), and the suprarenal IVC. The right-sided IVC (R) is patent while the left-sided IVC (L) with thrombus (stippled) empties into the left renal vein (lrv).

Furthermore, radiographic duplication of the IVC can be confused with saccular aortic aneurysms, aorto-lumbar lymphadenopathy, left pyeloureteric dilatation, retroperitoneal cysts, and loops of small bowel. [10] As a result it is imperative to consider this anomaly both in an acute presentation of venous thromboembolism in a younger individual, and with the above-mentioned disorders as well. A schematic diagram of the observed abnormality is shown in Figure 4.

As noted in our literature search, there were a few case reports of thromboembolic disease in patients with a duplicated IVC. In patients with DVT of the legs in this setting, the treatment paradigm protocol changes, caval interruption becomes paramount. The failure to interrupt both the right- and left-sided IVC can result in recurrent pulmonary embolism. [10] Physicians need to be reminded that such anomalies of the IVC exist and that they may influence decision-making in patients with an acute presentation of thromboembolic disease.

In all studies, age of presentation of first thrombosis has been less than 30 years of age and incidence is similar in men and women. Few studies [10-12] consider double inferior vena cava to be the cause of DVT, perhaps because it causes retrograde stasis less often. Compensatory drainage thru the thoracic-lumbar, pelvic, and abdominal veins can cause symptoms in the thorax, hypogastrium, lumbar, and genital regions, prior to those typical of DVT of the lower extremities. Early detection could warn of the presence of cava malformations in young patients. For instance our patient presented complaining of chest and abdominal pain, with associated discoloration of her left lower limb.

Some authors believe cava malformation alone can provoke DVT, [13] but the fact that lifelong asymptomatic malformations occur, [12] the findings in the case report and the status of thrombosis as a multifactorial illness, [14] suggest the presence of associated factors, both congenital and acquired. The complementary entities of the patient's heterozygosity for the Factor V Leiden mutation, and her use of an oral contraceptive intrauterine device, could invariably have been adjunctive triggers in her clotting cascade.

Ultimately there is currently no data available to guide the use of anticoagulation in the under 30 population who present with the additional complication of a congenital anomaly. Apparently, the most appropriate approach to treatment is for more than six months' anticoagulation while the principal factor provoking thrombosis continues. In conclusion, the possibility of recurrent thrombotic occlusion is high in these patients when anticoagulation treatment is withdrawn even when precipitating triggers such as oral contraceptives are removed.

## Consent

The patient has provided informed consent for the publication of this case report and accompanying images.

## Competing interests

The authors declare that they have no competing interests.

## Authors' contributions

The original manuscript was written by CM. All authors participated in drafting and editing the manuscript. All authors read and approved the final manuscript.

## Authors' information

The authors provide specialized, multidisciplinary clinical care for patients with a variety of hematologic and oncologic malignancies.
